# Supplementary material for: Bt Trait Efficacy Against Corn Earworm, Helicoverpa zea, (Lepidoptera: Noctuidae) for Preserving Grain Yield and Reducing Mycotoxin Contamination of Field Corn
Source: Insects. 2024 Nov 22;15(12):914. doi: 10.3390/insects15120914 (PMC11677160; doi:10.3390/insects15120914)
Supplement: Supplementary file 1 [file insects-15-00914-s001.zip › insects-3277268-supplementary.pdf]

Table S1. Effect of Bt traits on LS means ± SEM of percentage corn earworm infested ears and number of corn earworm larvae per ear by size and exit holes in R3 growth stage in 2019.

Brand & Hybrid	Bt Traits	Infested R3 ears (%)	Larvae by Size and Exit Holes per Ear				
			Small	Medium	Large	Exit	Total
DKC 6694	None (RR2)	55.0 ± 5.2 c	0.15 ± 0.06 cd	0.18 ± 0.09 b	0.15 ± 0.05 bc	0.22 ± 0.06 a	0.70 ± 0.10 bc
DKC 6697	Genuity VT Double PRO+	28.3 ± 4.7 e	0.16 ± 0.04 cd	0.10 ± 0.03 bc	0.04 ± 0.02 cd	0.02 ± 0.01 cd	0.32 ± 0.04 d
DKC 6629	Genuity Trecepta++	0 f	0 d	0 c	0 d	0 d	0 e
DKC 6205	None (RR2)	65.8 ± 6.1 b	0.15 ± 0.03 cd	0.19 ± 0.04 b	0.20 ± 0.04 b	0.22 ± 0.05 a	0.76 ± 0.06 b
DKC 6208	SmartStax+	41.7 ± 6.0 d	0.23 ± 0.07 c	0.11 ± 0.05 bc	0.10 ± 0.03 bcd	0.05 ± 0.02 bcd	0.49 ± 0.06 cd
P 1637R	None (RR2)	84.2 ± 5.2 a	0.57 ± 0.15 b	0.36 ± 0.05 a	0.45 ± 0.16 a	0.08 ± 0.05 b	1.46 ± 0.15 a
P 1637YHR	Optimum Intrasect+	80.8 ± 4.8 a	0.79 ± 0.15 a	0.33 ± 0.11 a	0.10 ± 0.05 bcd	0.07 ± 0.03 bc	1.30 ± 0.16 a
P 1637VYHR	Optimum Leptra++	0 f	0 d	0 c	0 d	0 d	0 e
P 2088R	None (RR2)	87.5 ± 5.4 a	0.57 ± 0.10 b	0.42 ± 0.08 a	0.38 ± 0.11 a	0.16 ± 0.06 a	1.52 ± 0.17 a
P 2089VYHR	Optimum Leptra++	1.7 ± 1.7 f	0.02 ± 0.02 d	0 c	0 d	0 d	0.02 ± 0.02 e
F > (*P*)							
Hybrid		<0.0001	<0.0001	<0.0001	<0.0001	<0.0001	<0.0001
Bt vs non-Bt		<0.0001	0.0009	<0.0001	<0.0001	<0.0001	<0.0001
Cry Bt vs non-Bt		<0.0001	0.0714	0.0829	<0.0001	<0.0001	0.0003
Vip Bt vs non-Bt		<0.0001	<0.0001	<0.0001	<0.0001	<0.0001	<0.0001
Cry Bt vs Vip Bt		<0.0001	<0.0001	<0.0001	0.0242	0.0116	<0.0001

LS means ± SEM within columns followed by the same letter are not significantly different (PROC MIXED, pair-wise t-tests of LSM, α = 0.05).
Hybrid: (df = 9, 54); Contrasting Statements: (df = 1, 54).

+ Bt-hybrid expressing only pyramided *Cry* proteins.

++ Bt-hybrid expressing pyramided *Cry* proteins with Vip3Aa20.

Table S2. Effect of Bt traits on LS means ± SEM of percentage corn earworm infested ears and number of corn earworm larvae per ear by size and exit holes in R3 growth stage in 2020.

Brand & Hybrid	Bt Traits	Infested R3 ears (%)	Larvae by Size and Exit Holes per Ear				
			Small	Medium	Large	Exit	Total
DKC 6694	None (RR2)	42.5 ± 14.3 cd	0.06 ± 0.03 ef	0.13 ± 0.07 bc	0.15 ± 0.05 ab	0.14 ± 0.06 b	0.48 ± 0.17 bcd
DKC 6697	Genuity VT Double PRO[+]	39.2 ± 13.6 d	0.18 ± 0.08 bcde	0.18 ± 0.07 abc	0.07 ± 0.04 bc	0 c	0.44 ± 0.16 cd
DKC 6629	Genuity Trecepta[++]	2.5 ± 2.5 e	0 f	0.01 ± 0.01 de	0.02 ± 0.02 c	0 c	0.02 ± 0.02 e
DKC 6205	None (RR2)	56.7 ± 10.8 ab	0.08 ± 0.03 def	0.11 ± 0.02 cde	0.16 ± 0.06 ab	0.22 ± 0.06 a	0.57 ± 0.11 abc
DKC 6208	SmartStax[+]	36.8 ± 13.4 d	0.20 ± 0.08 bcd	0.12 ± 0.07 cd	0.05 ± 0.03 c	0.02 ± 0.02 c	0.39 ± 0.15 d
DKC 6824	None (RR2)	55.0 ± 10.6 abc	0.12 ± 0.03 cdef	0.08 ± 0.02 cde	0.22 ± 0.08 a	0.17 ± 0.06 ab	0.60 ± 0.12 abc
DKC 6826	Genuity VT Double PRO[+]	37.5 ± 10.9 d	0.23 ± 0.09 bc	0.24 ± 0.09 ab	0.06 ± 0.03 c	0 c	0.53 ± 0.18 bcd
DKC 6799	Genuity Trecepta[++]	3.3 ± 3.3 e	0 f	0 e	0.01 ± 0.01 c	0.02 ± 0.02 c	0.03 ± 0.03 e
P 2088R	None (RR2)	49.2 ± 11.2 bcd	0.42 ± 0.11 a	0.16 ± 0.06 abc	0.06 ± 0.03 c	0.02 ± 0.01 c	0.65 ± 0.18 ab
P 2089VYHR	Optimum Leptra[++]	0 e	0 f	0 e	0 c	0 c	0 e
P 1637/1870R	None (RR2)	63.3 ± 11.1 a	0.22 ± 0.10 bc	0.19 ± 0.05 abc	0.20 ± 0.09 a	0.12 ± 0.03 b	0.73 ± 0.14 a
P 1637/1870YHR	Optimum Intrasect[+]	54.2 ± 10.9 abc	0.30 ± 0.08 ab	0.25 ± 0.12 a	0.04 ± 0.02 c	0.04 ± 0.01 c	0.63 ± 0.14 ab
F > (*P*)							
Hybrid		<0.0001	0.0002	0.0295	0.0058	<0.0001	<0.0001
Bt vs non-Bt		<0.0001	0.0630	0.3788	<0.0001	<0.0001	<0.0001
Cry Bt vs non-Bt		0.0003	0.1269	0.0218	<0.0001	<0.0001	0.0123
Vip Bt vs non-Bt		<0.0001	<0.0001	0.0003	<0.0001	<0.0001	<0.0001
Cry Bt vs Vip Bt		<0.0001	<0.0001	<0.0001	0.0492	0.6653	<0.0001

LS means ± SEM within columns followed by the same letter are not significantly different (PROC MIXED, pair-wise t-tests of LSM, α = 0.05).
Hybrid: (df = 11, 66); Contrasting Statements: (df = 1, 66).
[+] Bt-hybrid expressing only pyramided *Cry* proteins.
[++] Bt-hybrid expressing pyramided *Cry* proteins with Vip3Aa20.

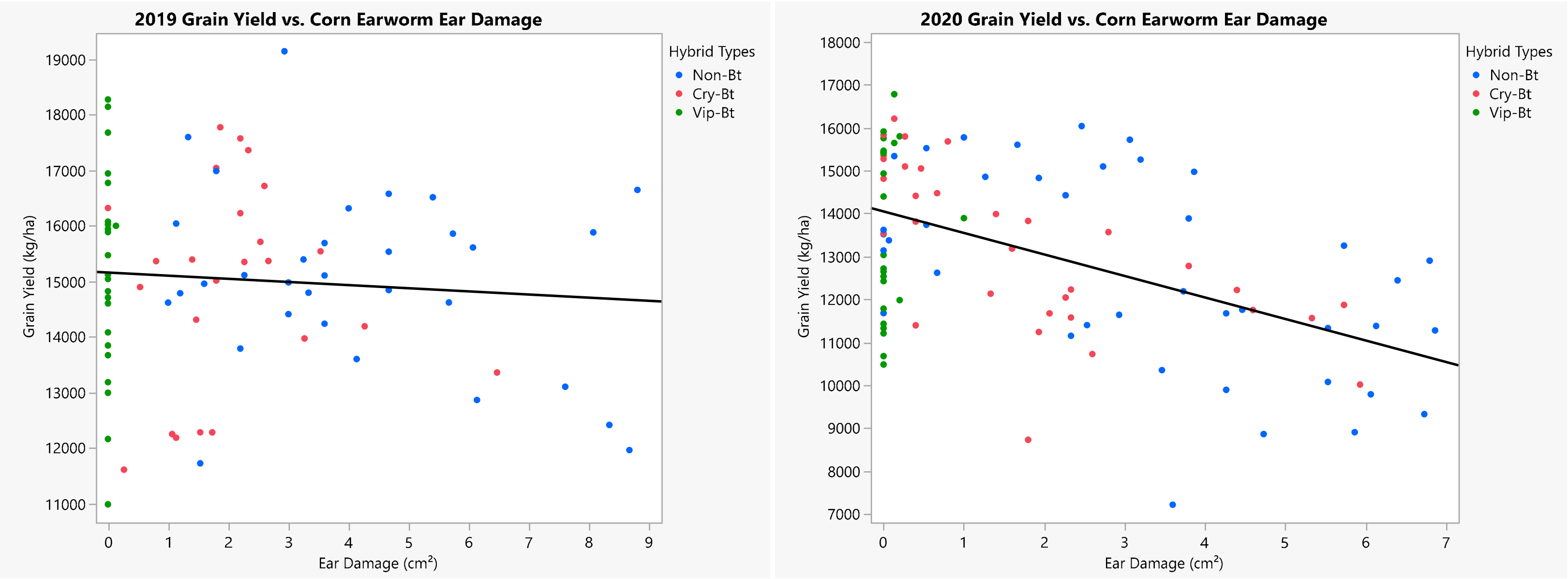

Supplemental Figure S1. Linear regression analysis depicting the relationship between corn earworm ear damage and grain yield of field corn by year. 2019: $R^2 = 0.0061$; $F = 0.4791$; $P = 0.4909$. 2020: $R^2 = 0.2638$; $F = 33.690$; $P < 0.0001$.
